# Predictive Value of Excision Repair Cross Complementation Group 1 (ERCC1) by Immunohistochemistry for Determining Neoadjuvant Chemotherapy Response in Triple-Negative Breast Cancers

**DOI:** 10.1155/tbj/8410670

**Published:** 2025-02-18

**Authors:** Atif Ali Hashmi, Yumna Ajaz, Muhsana Sajjad, Fazail Zia, Muhammad Irfan, Syed Muhammad Abu Bakar, Erum Yousuf Khan, Naveen Faridi

**Affiliations:** ^1^Department of Histopathology, Liaquat National Hospital and Medical College, Karachi, Pakistan; ^2^Department of Pathology, Memorial Sloan Kettering Cancer Center, New York City, New York, USA; ^3^Department of Pathology, Liaquat National Hospital and Medical College, Karachi, Pakistan; ^4^Department of Pathology, Jinnah Sindh Medical University, Karachi, Pakistan; ^5^Department of Statistics, Liaquat National Hospital and Medical College, Karachi, Pakistan

**Keywords:** excision repair cross complementation group 1 (ERCC1), neoadjuvant chemotherapy, pathological complete response, platinum-based chemotherapy, triple-negative breast cancer (TNBC)

## Abstract

**Introduction:** Triple-negative breast cancers (TNBCs) constitute a significant proportion of breast cancers in Pakistan. Owing to the lack of expression of hormone (estrogen/progesterone) receptor and human epidermal growth factor receptor 2 (HER2neu), treatment options for TNBCs are limited. Therefore, it is important to identify markers that predict response to chemotherapy in these patients. Previous studies have demonstrated that the excision repair cross complementation group 1 (ERCC1) protein can successfully augur the response to chemotherapy in cancer; however, data related to TNBCs, particularly in Pakistan, are limited. Therefore, in this study, we evaluated the role of ERCC1 in predicting the response to neoadjuvant chemotherapy in patients with TNBCs.

**Methods:** This cross-sectional study was conducted at the Liaquat National Hospital, Histopathology Department, between January 2019 and June 2023. A total of 132 biopsy-proven cases of breast cancer that were negative for estrogen receptor (ER), progesterone receptor (PR), and HER/2neu and were administered neoadjuvant chemotherapy before surgery were included in the study. ERCC1 immunohistochemical (IHC) staining was performed on prechemotherapy needle biopsies. The results were scored semiquantitatively by assessing the average intensity on a scale of 0–3 (0, no staining; 1, weak nuclear staining; 2, intermediate nuclear staining; and 3, strong nuclear staining) and the proportion of tumor cells showing positive nuclear staining. The intensity and proportion scores were then multiplied to give a score that was divided by 100 to give an overall score, and scores equal to or higher than 1.0 were considered positive. Neoadjuvant chemotherapy response was categorized as pathological complete response (pCR) when no residual invasive breast carcinoma was found on the postneoadjuvant chemotherapy excision specimen and as pathological partial response (pPR) when residual cancer cells were present in admixed chemotherapy-related changes. The residual cancer burden (RCB) was calculated using the MD Anderson RCB calculator. The association between ERCC1 expression and the chemotherapy response/RCB class was determined.

**Results:** We found that 90.9% (*n* = 120) of TNBC cases expressed ERCC1, whereas pCR was noted in 24 (18.2%) cases. A significant association was observed between ERCC1 expression and pCR. Cases with negative ERCC1 expression had a significantly higher frequency of pCR (66.7%) than those with positive ERCC1 expression (13.3%). Additionally, the ERCC1-positive group showed a higher frequency of RCB classes II (36.7%) and III (43.3%) than the ERCC1-negative group (RCB II: 25%; RCB III: 0%). Moreover, positive ERCC1 expression was associated with higher nodal (N) stage.

**Conclusion:** In this study, we established the role of negative ERCC1 expression in predicting the response to chemotherapy in neoadjuvant TNBC. Therefore, ERCC1 can be used as a predictive marker to stratify patients who will benefit from neoadjuvant therapy. Moreover, we also noted an association between ERCC1 expression and nodal metastasis; however, more large-scale studies are needed to establish its role as a prognostic biomarker in TNBC.

## 1. Introduction

Triple-negative breast cancer (TNBC) accounts for approximately 16% of all breast cancers and is characterized by the lack of expression of estrogen receptor (ER), progesterone receptor (PR), and human epidermal growth factor receptor 2 (HER2neu) [1, 2]. Histologically, TNBCs show varied morphology; most are invasive breast carcinomas, no special type (IBCs-NST); however, a significant number of cases show metaplastic and medullary-like morphology [2]. They are typically of high grade and show poor overall survival and an increased risk of recurrence [1, 3]. Moreover, due to the lack of expression of hormone receptors and HER2neu, TNBCs do not benefit from tamoxifen and trastuzumab.

Complete surgical resection in the form of breast conservation surgery (BCS) or mastectomy is the mainstay of treatment for TNBCs; however, neoadjuvant chemotherapy is recommended for locally advanced cases. Neoadjuvant chemotherapy is also recommended for early-stage cancers, up to tumor (T) stage T1c [4]. The pathological response to neoadjuvant chemotherapy is one of the most important parameters determining prognosis in these patients [5]. Studies exploring pathological predictors of response to neoadjuvant chemotherapy have found high Ki67 and PDL1 expression and abundance of tumor-infiltrating lymphocytes to predict pathological complete response (pCR) [6, 7].

Platinum-based chemotherapy is commonly used to treat TNBCs. The tumor-killing effect of platinum-based chemotherapy is attributed to the formation of DNA adducts that inhibit DNA replication. Excision repair cross-complementation 1 (ERCC1) is an important gene, and its protein product plays a crucial role in the nucleotide excision repair (NER) pathway as it creates a heterodimeric complex with xeroderma pigmentosum complementation group F (XPF) protein, which then performs the function of DNA excision. An intact NER pathway confers resistance to platinum-based chemotherapy. Increased ERCC1 expression is associated with resistance to platinum-based chemotherapy in various body tumors [8–10]. The predictive role of ERCC1 expression in determining the chemotherapy response in TNBCs has not been widely studied. The two main parameters that determine the chemotherapy response include residual cancer burden (RCB) score/class and pCR as determined by histological examination of the surgical breast specimen. Therefore, in this study, we evaluated the association between ERCC1 expression and neoadjuvant chemotherapy response in patients with TNBCs.

## 2. Methods

### 2.1. Patients and Methods

This retrospective cross-sectional study was conducted at the Department of Histopathology of Liaquat National Hospital between January 2019 and June 2023. Clinical data for this study were collected from relevant patient files obtained from hospital records. Ethical approval for the study was taken from the Ethical Review Committee, Liaquat National Hospital (App#0968-2023-LNH-ERC). Among these data, 132 cases of breast cancers that underwent platinum-based neoadjuvant chemotherapy prior to surgery and were confirmed via biopsy to be negative for ER, PR, and HER2neu were included in the study. Any patient file that was not complete with the required clinical data automatically met our exclusion criteria. Similarly, if any biomarker study was also not conducted in any of TNBC cases, then that case was also excluded from our data pool. Histopathological tumor features, including tumor type and grade, were evaluated on prechemotherapy core-needle biopsy specimens.

### 2.2. ERCC1 Immunohistochemical (IHC) Analysis

**Figure 1:**
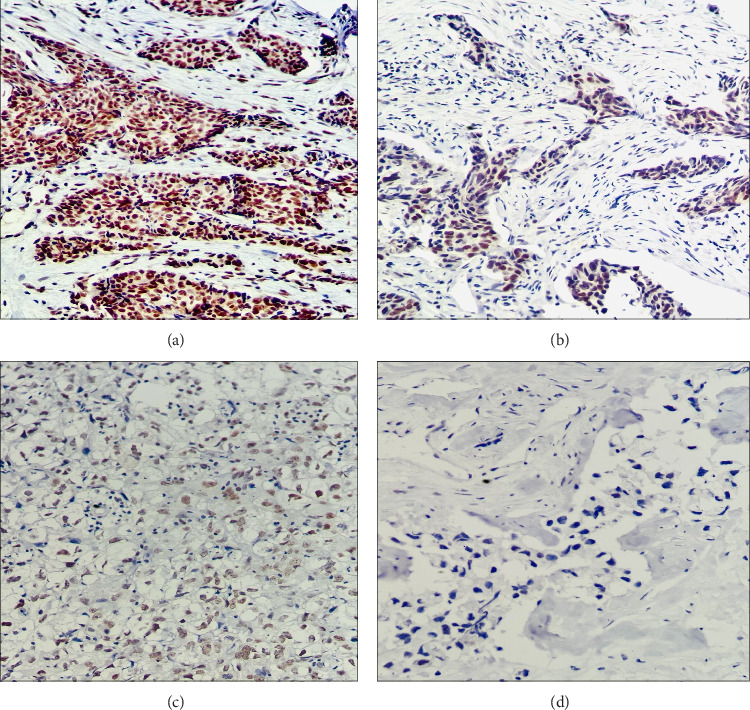
Immunohistochemical expression of ERCC1 in triple-negative breast cancer. (a) Strong nuclear expression of ERCC1. (b) Intermediate nuclear staining of ERCC1 in cancer cells. (c) Weak nuclear expression of ERCC1. (d) No nuclear staining of ERCC1 in cancer cells.

ERCC1 IHC staining was performed on prechemotherapy needle biopsies. The results were scored semiquantitatively by assessing the average intensity on a scale of 0–3 (0, no staining; 1, weak nuclear staining; 2, intermediate nuclear staining; and 3, strong nuclear staining) and the proportion of tumor cells showing positive nuclear staining (Figure 1).

The intensity and proportion scores were then multiplied to give a score that is divided by 100 to give an overall score, and scores equal to or higher than 1.0 were considered positive [11]. Ki67 IHC was also applied during prechemotherapy, and the results are presented as percentages of positively stained cancer cells.

### 2.3. Neoadjuvant Chemotherapy Response Categorization

All patients received platinum-based neoadjuvant chemotherapy before definite surgical resection. Definite surgical resection (mastectomy, modified radical mastectomy, or BCS) was performed after the completion of chemotherapy, and specimens were sent to the histopathology laboratory. Neoadjuvant chemotherapy response was assessed histopathologically and categorized as pCR when no residual IBC was found on postneoadjuvant chemotherapy excision specimen and as pathological partial response (pPR) when residual cancer cells were present in admixed chemotherapy-related changes. The RCB score was calculated using MD Anderson RCB calculator (https://www.mdanderson.org/breastCancer_RCB). Furthermore, RCB class categorization was performed. Cases with no residual cancer cells, equivalent to pCR, were labeled as RCB 0. Cases with an RCB > 0 but ≤ 1.36 were categorized as RCB II. Cases with an RCB > 1.36 but ≤ 3.28 were called as RCB II. RCB III was labeled when the RCB score was > 3.28 [11].

### 2.4. Statistical Analysis

**Figure 2:**
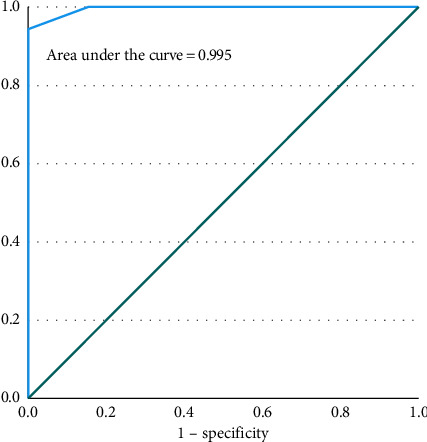
ROC analysis for prediction of ERCC1 expression.

Data analysis was performed using the Statistical Package for Social Sciences (Version 26.0, IBM Corp., Armonk, NY, USA). The chi-square test, independent *t*-test, and Fisher's exact test were used to check the association between ERCC1 expression and various clinicopathological parameters. Odds were calculated by logistic regression. Area under curve was obtained by receiver operating characteristic (ROC) curve, and predictive values were obtained by Youden index. The area under the curve indicates that the score (AUC = 0.995) is the good predictor of ERCC1.  Figure 2 display the ROC curve along with area under the curve. In terms of the detection of ERCC1, the optimal cutoffs were 1.00 (sensitivity = 94.2%, specificity = 100%), 0.85 (sensitivity = 100%, specificity = 83.3%), and 0.65 (specificity = 100%, specificity = 75%).  Table 1 displays the detailed results of the optimal cutoff for the scores as well as the values of the Youden index. *p* values < 0.05 were considered significant.

## 3. Results

### 3.1. Clinicopathological Characteristics of Study Population

There were 132 breast cancer samples included in our study. The demographic and clinical characteristics of the study population are presented in Table 2. The mean age of patients was found to be 46.39 ± 11.06 years, with a majority (66.7%) of patients falling in the age group of 36–50 years. The mean tumor size and Ki67 index were found to be 4.81 ± 2.26 cm and 47.53 ± 22.55, respectively. Histologically, 116 out of 132 (87.9%) cases comprised IBS-NST. Approximately half (54.5%) of the tumors were Grade 3 tumors before chemotherapy. Most cases (48.5%) were Stage T2. Nodal metastasis was found in 78.8% of the cases. In our study, we found that 81.8% of cases had a pPR to chemotherapy, whereas only 18.2% had a pCR. Moreover, most tumors showed a high RCB, as evidenced by the RCB categorization (RCB II: 35.6%; RCB III: 39.4%), as shown in Table 3.

### 3.2. Association of ERCC1 Expression With Clinicopathological Parameters

Table 1 shows the association between clinicopathological parameters and ERCC1 expression. A significant association was noted between ERCC1 expression and age and nodal stage. The negative expression of ERRC1 was significantly higher in the age group of 36–50 years (*p*=0.036). Moreover, positive ERCC1 expression was significantly associated with a higher nodal stage (*p* < 0.001). No significant association was observed between ERCC1 expression and tumor size, grade, Ki67 index, or T stage (Table 3).

### 3.3. Association of (ERCC1 Expression With Neoadjuvant Chemotherapy Response

**Figure 3:**
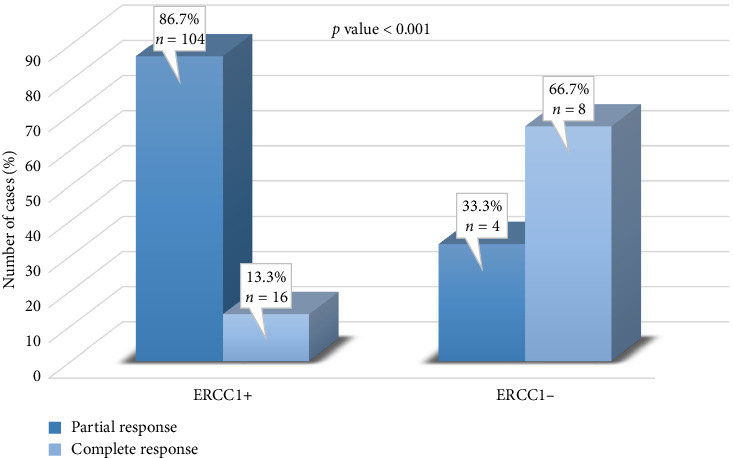
Association of ERCC1 expression with pathological response to neoadjuvant chemotherapy in triple-negative breast cancer.

Figure 3 shows the association between ERCC1 expression and neoadjuvant chemotherapy response. A significant association was observed between ERCC1 expression and pCR. Cases with negative ERCC1 expression had a significantly higher frequency of pCR (66.7%) than those with positive ERCC1 expression (13.3%).

**Figure 4:**
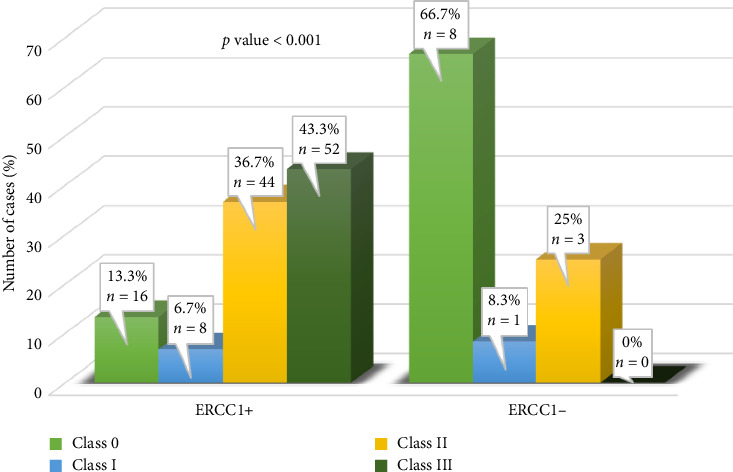
Association of ERCC1 expression with residual cancer burden (RCB) class in triple-negative breast cancer.

Figure 4 illustrates the association between ERCC1 expression and the RCB class. The ERCC1-positive group showed a higher frequency of RCB classes II (36.7%) and III (43.3%) than the ERCC1-negative group (RCB II: 25%; RCB III: 0%).

Table 4 shows the association of ERCC1 expression with clinicopathological parameters by univariate and multivariate analyses. ERCC1-positive cases show a lower likelihood of N0 stage, pCR, and lower RCB class (Class 0/I).

## 4. Discussion

In this study, we found that negative ERCC1 expression was associated with a higher frequency of pCR and a low RCB. This indicates that ERCC1 expression confers resistance to platinum-based chemotherapy in TNBCs. Moreover, we also noted that ERCC1 expression was associated with a higher nodal stage, indicating its prognostic relevance in TNBCs.

A few studies have evaluated the prognostic and predictive values of ERCC1 expression in TNBCs. El Baiomy and El Kashef, in a study involving 52 patients with metastatic TNBCs, found that ERCC1 expression by immunohistochemistry was associated with poor progression-free survival and platinum-based chemotherapy response rates [12]. Our findings are concordant with those of this study; however, survival was not assessed.

The expression rate of ERCC1 in TNBCs also varies, indicating genetic variability. Ozkan et al. conducted a study involving 45 TNBC samples and found that ERCC1 expression was 73.3%. They used a semiquantitative ERCC1 scoring approach similar to that used in our study [13]. In our study, ERCC1 expression was > 90%. Therefore, large-scale studies are needed to determine the approximate proportion of TNBCs in each population that expresses ERCC1. This approach may help guide planning guidelines regarding whether to incorporate ERCC1 testing in TNBCs as a routine or not.

In our study, we noted that ERCC1 expression was associated with a higher nodal stage. Nodal metastasis is one of the most important prognostic parameters in breast cancer. Conversely, a few studies found that ERCC1 expression was associated with prognostic parameters. Gerhard et al. found that ERCC1 expression was associated with smaller tumor size. They also found that TNBCs were more likely to be ERCC1-negative than non-TNBCs. Interestingly, ERCC1 negativity in their study was quite high compared with that in the rest of the literature (61.5%) [14]. The findings may be attributed to a different interpretation of IHC staining. Therefore, a standard IHC approach/guideline should be established. Abdel-Fatah et al. highly advocated ERCC1 evaluation in patients with breast cancer because of their large cohort. They noted that in locally advanced breast cancers, low ERCC1 expression was associated with high pCR and low distant relapse rates. They also noted that the pCR rate differs between ER-positive and ER-negative breast cancers [15]. These findings are consistent with our results; however, our cohort did not include hormone receptor positive cases.

ERCC1 is a key gene involved in the NER . Studies have demonstrated its prognostic value in many human cancers, including cervical, lung, and bladder cancers, especially in advanced stages [16–18]. ERCC1 has both prognostic and predictive values in breast cancer; however, previous studies did not establish an association between ERCC1 expression and tumor type or grade. Conversely, an association between ERCC1 positivity and ER expression was noted. ER-positive tumors express higher levels of ERCC1 [14]. The prognostic role of ERCC1 was demonstrated in previous studies. A study by Li, Liao, and Ma proved the poor prognostic value of ERCC1 in patients with HER2neu-expressing breast cancer, as evidenced by lower disease-specific survival and overall survival in ERCC1-positive patients [19]. Similarly, a study showed that ERCC1-positive TNBCs have a poor prognosis [20]. In addition to the prognostic value, the predictive significance of ERCC1 was previously studied. A study involving 52 patients with metastatic TNBCs suggested the inclusion of ERCC1 expression in planning treatment options for advanced TNBCs. Because ERCC1-positive tumors exhibit resistance to cisplatin, the exclusion of cisplatin-based chemotherapy can minimize its toxic effects in this group of patients [12].

In summary, ERCC1 positivity confers resistance to neoadjuvant chemotherapy in TNBCs as evidenced by a lower frequency of pCR and higher RCB scores in ERCC1-positive cases. Moreover, we also found a poor prognostic parameter associated with ERCC1 positivity, which is higher nodal stage. The possible mechanism behind the association of ERCC1 expression and chemotherapy resistance is as follows: Alkylating agents and platinum-based drugs basically cause tumor cell death by DNA damage and formation of DNA adducts, and one of the basic defense mechanisms against this DNA damage possessed by the cancer cells is DNA repair. ERCC1 is one of the key DNA repair enzymes. ERCC1-expressing cancer cells have an ability to repair the DNA damage caused by platinum-based drugs and survive [21]. Our study uncovers a very important aspect of neoadjuvant chemotherapy resistance in TNBCs; however, these findings need to be further uncovered at a molecular level.

### 4.1. Limitations

Our study has a few limitations. We only included TNBCs; therefore, the pCR rate in ER-positive breast cancers was not evaluated. Moreover, given that this was a single-center study, the results cannot be generalized to the entire population, necessitating the need for large-scale multicenter studies. Furthermore, we did not assess the association between ERCC1 expression and overall survival or disease-free survival because long-term follow-up data were not available. Finally, molecular studies were not conducted to link gene expression with IHC expression of ERCC1.

## 5. Conclusions

We found that ERCC1 is a promising IHC marker in TNBCs because of its predictive and prognostic relevance. We noted that ERCC1 expression was associated with a lower pCR and a higher RCB in neoadjuvant TNBC, indicating that ERCC1 confers chemotherapy resistance in TNBCs. Furthermore, ERCC1 expression was associated with a higher nodal stage, indicating poor prognosis in TNBCs. Future studies are recommended to further evaluate the association between ERCC1 expression and disease-free survival in patients with TNBCs.

## Figures and Tables

**Table 1:** Association of excision repair cross complementation group 1 (ERCC1) expression with clinicopathological parameters.

Clinicopathological parameters	Values		*p* value	Effect size
	ERCC1 expression			
	Positive	Negative		
Age group⁣^∗^				
≤ 35 years, *n* (%)	16 (13.3)	0 (0)	0.036⁣^∗∗^	
36–50 years, *n* (%)	76 (63.3)	12 (100)		0.115
> 50 years, *n* (%)	28 (23.3)	0 (0)		
Tumor size (cm)⁣^∗^				
≤ 5 cm, *n* (%)	76 (63.3)	8 (66.7)	1.000	0.020
> 5 cm, *n* (%)	44 (36.7)	4 (33.3)		
Ki67 group (*n* = 112)⁣^∗^				
< 15%, *n* (%)	8 (8)	0 (0)	0.145	
15%–25%, *n* (%)	12 (12)	0 (0)		0.037
26%–50%, *n* (%)	32 (32)	8 (66.7)		
> 50%, *n* (%)	48 (48)	4 (33.3)		
RCB score, mean ± SD	2.99 ± 1.74	0.58 ± 0.95	< 0.001⁣^∗∗^	0.365
Histological subtype⁣^∗^				
Invasive breast carcinoma, NST, *n* (%)	104 (86.7)	12 (100)	0.359	0.117
Others, *n* (%)	16 (13.3)	0 (0)		
Tumor (T) stage⁣^∗^				
T1, *n* (%)	20 (16.7)	0 (0)	0.271	
T2, *n* (%)	56 (46.7)	8 (66.7)		0.020
T3/T4, *n* (%)	44 (36.7)	4 (33.3)		
Nodal (N) stage⁣^∗^				
N0, *n* (%)	20 (16.7)	8 (66.7)	< 0.001⁣^∗∗^	
N1, *n* (%)	44 (36.7)	4 (33.3)		
N2, *n* (%)	46 (38.3)	0 (0)		0.352
N3, *n* (%)	10 (8.3)	0 (0)		
Tumor grade⁣^∗^				
Grade 1, *n* (%)	20 (16.7)	0 (0)	0.370	
Grade 2, *n* (%)	36 (30)	4 (33.3)		0.077
Grade 3, *n* (%)	64 (53.3)	8 (66.7)		

Abbreviations: NST, no special type; RCB, residual cancer burden.

⁣^∗^Fisher's exact test was applied.

⁣^∗∗^*p* value significant at < 0.05.

**Table 2:** Optimal cutoff for detection of ERCC1.

Positive if ≥	Sensitivity (%)	Specificity (%)	Youden index
1.00	94.2	100	0.942
0.85	100	83.3	0.833
0.65	100	75.0	0.75

**Table 3:** Clinicopathological parameters of population under study.

Clinicopathological parameters	Values
Age (years), mean ± SD	46.39 ± 11.06
Age groups	
≤ 35 years, *n* (%)	16 (12.1)
36–50 years, *n* (%)	88 (66.7)
> 50 years, *n* (%)	28 (21.2)
Tumor size (cm), mean ± SD	4.81 ± 2.26
Tumor size	
≤ 5 cm, *n* (%)	84 (63.6)
> 5 cm, *n* (%)	48 (36.4)
Ki67 (%), mean ± SD (*n* = 112)	47.53 ± 22.55
Ki67 groups (*n* = 112)	
< 15%, *n* (%)	8 (7.1)
15%–25%, *n* (%)	12 (10.7)
26%–50%, *n* (%)	40 (35.7)
> 50%, *n* (%)	52 (46.4)
RBC score, mean ± SD	2.77 ± 1.82
RCB class	
Class 0, *n* (%)	24 (18.2)
Class I, *n* (%)	9 (6.8)
Class II, *n* (%)	47 (35.6)
Class III, *n* (%)	52 (39.4)
Histological type	
Invasive breast carcinoma, NST, *n* (%)	116 (87.9)
Others, *n* (%)	16 (12.1)
Tumor (T) stage	
T1, *n* (%)	20 (15.2)
T2, *n* (%)	64 (48.5)
T3/T4, *n* (%)	48 (36.4)
Nodal (N) stage	
N0, *n* (%)	28 (21.2)
N1, *n* (%)	48 (36.4)
N2, *n* (%)	46 (34.8)
N3, *n* (%)	10 (7.6)
Procedure	
BCS, *n* (%)	44 (33.3)
MRM, *n* (%)	64 (48.5)
Simple mastectomy, *n* (%)	24 (18.2)
Laterality	
Right, *n* (%)	76 (57.6)
Left, *n* (%)	56 (42.4)
Tumor grade	
Grade 1, *n* (%)	20 (15.2)
Grade 2, *n* (%)	40 (30.3)
Grade 3, *n* (%)	72 (54.5)
Neoadjuvant chemotherapy response	
Partial response, *n* (%)	108 (81.8)
Complete response, *n* (%)	24 (18.2)
ERCC1 expression	
Positive, *n* (%)	120 (90.9)
Negative, *n* (%)	12 (9.1)

Abbreviations: BCS, breast conservation surgery; ERCC1, excision repair cross complementation group 1; MRM, modified radical mastectomy; NST, no special type; SD: standard deviation.

**Table 4:** Association of clinicopathological parameters with ERCC1 expression: univariate and multivariate analyses.

Clinicopathological parameters	Unadjusted		Adjusted	
	OR (95% CI)	*p* value	OR (95% CI)	*p* value
Age group⁣^∗^				
≤ 45 years, *n* (%)	0.438 (0.125–1.531)	0.196	—	—
> 45 years, *n* (%)	1.000	—	—	—
Tumor size (cm)⁣^∗^				
≤ 5 cm, *n* (%)	0.864 (0.246–3.034)	0.819	—	—
> 5 cm, *n* (%)	1.000	—	—	—
Ki67 group (*n* = 112)⁣^∗^				
≤ 30%, *n* (%)	0.778 (0.217–2.789)	0.700	—	—
> 30%, *n* (%)	1.000	—	—	—
RBC classification				
Class 0/Class I	0.083 (0.021–0.332)	< 0.001⁣^∗^	0.108 (0.025–0.457)	0.003⁣^∗^
Class II/Class III	1.000	—	1.000	—
Histological subtype⁣^∗^				
Invasive breast carcinoma, NST, *n* (%)	NA	0.998	—	—
Others, *n* (%)	1.000	—	—	—
Tumor (T) stage⁣^∗^				
T1/T2, *n* (%)	0.864 (0.246–3.034)	0.819	—	—
T3/T4, *n* (%)	1.000		—	—
Nodal (N) stage⁣^∗^				
N0, *n* (%)	0.100 (0.027–0.364)	< 0.001⁣^∗^	0.134 (0.034–0.532)	0.004⁣^∗^
N1–N3, *n* (%)	1.000	—	1.000	—
Tumor grade⁣^∗^				
Grade 1/Grade 2, *n* (%)	1.750 (0.500–6.124)	0.381	—	—
Grade 3, *n* (%)	1.000	—	—	—
Chemotherapy response				
Complete response, *n* (%)	0.077 (0.021–0.285)	< 0.001⁣^∗^	0.126 (0.024–0.659)	0.002⁣^∗^
Partial response, *n* (%)	1.000	—	1.000	—

*Note:* Binary logistics regression was applied.

⁣^∗^Significant at 0.05 level.

## Data Availability

Data file is available on request. Please contact the author A.A.H. (atifhashmi345@gmail.com) for data requests.
